# Adénocarcinome ano-réctal après traitement par Infliximab pour une maladie de Crohn fistulisante

**DOI:** 10.11604/pamj.2017.26.172.10218

**Published:** 2017-03-24

**Authors:** Jihane Smaali, Youssef Sekkach

**Affiliations:** 1 Département de Médecine Interne, Hôpital Militaire d'Instruction Mohammed V, Université Med V, Souissi, Rabat, Maroc

**Keywords:** Maladie de Crohn, infliximab, adénocarcinome ano-réctal, Crohn disease, infliximab, anorectal adenocarcinoma

## Image en médecine

Patient de 35 ans, suivi pour une maladie de Crohn anopérinéale traitée par corticoides puis, 6-mercaptopurine et azathioprine, avec une rémission prolongée. Durant l'évolution, le patient présenta une altération rapide de l'état général. Les investigations morphologiques étaient en faveur d'une poussée grave dans le cadre de sa maladie. Le diagnostic d'une maladie de Crohn ano-périnéale dans sa forme fistulisante était posé (A), et le patient avait bénéficiée d'une antibiothérapie à large spectre, une alimentation parentérale, une mise au repos du tube digestif et une colostomie de décharge ayant permis une amélioration clinique, puis la mise sous anti-TNF alpha type Infliximab à la dose de 5mg/kg (S0, S2, S4 et S8). L'évolution s'est compliquée de l'apparition progressive d'une lésion anale d'allure tumorale (B) augmentant progressivement de volume avec altération rapide de l'état général. Une nouvelle imagerie abdominale (IRM) permettait d'individualiser un processus ano-réctal d'allure tumorale, immunohistochimiquement en faveur d'un adénocarcinome ano-rectal infiltrant. 4,3% de cas d'adénocarcinome ano-rectaux sur maladie de crohn fitulisante ont été rapportés. A la lumière de cette illustration, nous accordons une attention très particulière sur la possible transformation maligne chez tout patient traité par Infliximab pour une maladie de Crohn fistulisante, et nous insistons sur une évaluation histologique de toute lésion anale ou périnéale avant traitement par anti-TNF. Le diagnostic reste difficile en raison des phénomènes inflammatoires et / ou des sténoses concomitantes; Les moyens d'imagerie, y compris les examens TDM et l'IRM semblent avoir une faible sensibilité dans la détection du cancer.

**Figure 1:**
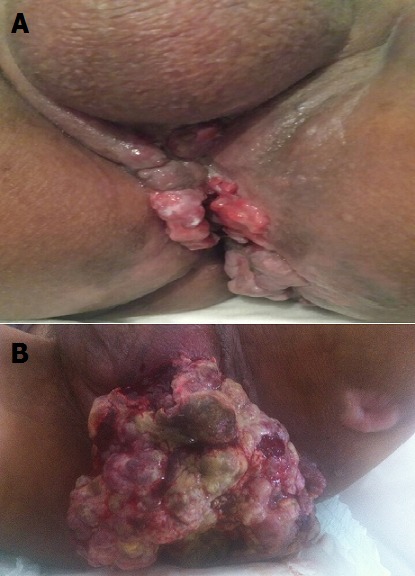
A) maladie de Crohn fistulisante avant infliximab; B) maladie de crohn fistulisante après infliximab

